# Unstable Mental Equilibrium

**Published:** 1890-03-15

**Authors:** 


### The Doctor's Pulpit.

### UNSTABLE MENTAL EQUILIBRIUM.

To an inhabitant of a strange planet where drunkenness has never been known a drunken man would present a peculiar spectacle. Let it be assumed that the stranger is, like ourselves, possessed of an upright body, legs, arms, a back-bone, manifold sets of muscles, and a brain and spinal cord, in all respects similar to our own. The men and women of his particular planet, with all their ways of walking, running, leaping, sitting, standing, riding, and so on, will be perfectly familiar to him. But he has never in his whole life seen one of them under the influence of drink. Strolling about on our earth, in town or country, he suddenly becomes aware of an extraordinary phenomenon. Coming towards him is a creature that looks like a man. But there is something most unusual about his legs: they seem as if they only partly belong to him, and were trying to turn off at various tangents on their own account. The creature heels over to the right, and making a desperate effort to recover himself, plunges recklessly to the left. All of a sudden he dashes forward, and with equal abruptness pulls himself up and endeavours to stand firmly on his disloyal legs. He sees a lamp-post near, and for some reason best known to himself appears amiably desirous of shaking hands with it. Finally a more frantic lurch than usual carries him to the ground, where he lies more or less happy until his nerves and muscles recover the power of maintaining him in stable equilibrium.

It would not be easy to imagine anything which would better illustrate the ideas expressed by the words "futility," "absurdity," "purposelessness," and "waste" than the drunken man just pictured. What the visitor from the strange planet would think or say we can only guess at. Our present purpose is not to deal with drunkenness, but simply to use the drunken man as an illustration which will help us to understand what is meant by "unstable mental equilibrium."

Most of the people who live in the world have some kind of value. Where should we find a man who, if asked, would admit that he was of no more worth than an old rag? Some human beings have a brain value which cannot be computed by money or any other standard. It is a question of surpassing importance to each individual whether he and his family and the world get out of his brain power all that they ought to get. Recurring to our drunken man for comparison we find that whilst in a state of drunkenness he is not only worthless, but worse than worthless both to himself and to all who have any concern with him. A question which experience and observation have suggested to the writer is this: has the average man any habitual lapses from mental equilibrium analogous to the drunkard's lapses from physical equilibrium? And do those lapses prevent a man from accomplishing that work in life which he has it in him to accomplish, exactly as the drunkard's lapses interfere with his business and social success? In other words, is it possible for the mental backbone to lose its erectness, for the mental legs to "wobble," and for the mental muscles to become paralysed and powerless, so that the whole mind, which is the whole man, flounders aimlessly about or falls prone upon the ground in motionless imbecility? It is undoubtedly possible! Many persons have habitually as little mental steadiness and purpose as the habitual drunkard has of bodily equability.

Some who read this and think of it will be able to put their fingers upon quite a number of their neighbours and friends who, as they think, indulge in this kind of mental intoxication. But careful observation seems to show that all people, with very few exceptions, bring at least partial failure upon their projects and purposes by their frequent loss of mental equilibrium. It is a comparatively rare thing for human beings to have in early life greatness of mind enough to form a great life purpose. It is still more rare to find a man who, having formed such a purpose early in life, carries it out steadily, intelligently, and resolutely to the very last day of his earthly existence. Perhaps it is not too much to say that no man who has done this has ever failed to come to some kind of greatness before his death. It is this very want of intelligent, steadfast, and persistent purpose in individual men which has been the parent of most of the miseries, social, political, and religious, that have afflicted the world.

The aspect of the question which chiefly affects us at the present time is the condition of our own particular epoch, and of those of us who live in it. The doctrine of development has something to say on this point. We may develop in what direction we will, but if individuals do not develop in strength and intelligence of purpose nothing can save either single persons or masses of men from signal catastrophe. "Am I," each man may ask, "and are my neighbours, always mentally erect and sober? Or am I, and are many of them, as destitute of the power to move steadily forward in a purposed straight line as the drunkard is to keep to the line of a foot-wide pavement? If not, what are the mental intoxicants that impair and destroy my equilibrium?"

That mental intoxication and consequent mental powerlessness are common in these days is very evident to the observant physiologist. All classes of men do their routine work very well: but where can a man of original mind be found? If wheat be sown in a field, the rain may fall, the sun may shine, top-dressings of several kinds may be thrown upon it, and even clover seeds may be sown among it without injury and with some advantage to the original wheat crop. But however excellent the original seed of the wheat may have been, and however strong a hold it may have taken upon the soil, if barley, oats, tares, rye-grass, clover, and twenty other different kinds of seeds, all of which may be excellent in themselves, be sown in it, the original wheat must be almost entirely destroyed and the final crop can only be a tangled mass, worthless both for man and beast. This gives us a picture of what often happens in these times. Men, most of whom in childhood have some little originality of mind, are allowed no free play for the development of their natural individuality. The schoolmaster takes the first turn at the work of choking the natural crop with too many kinds of seed. The College professor in many cases makes the business complete. What school and college have failed to accomplish the writer of books tries his skill upon. So that by the time the average man has reached thirty he is for all practical purposes as destitute of mental backbone as the drunkard is of physical uprightness; and he plunges about in the regions of intellect and ideas as aimlessly, as stupidly, and with as little result as the drunkard who falls prone in the street. Will men never learn that a man's own original mind is the seed which is designed to come to a crop, and that other men's ideas should be employed upon it as top-dressings, or weedings, or other kinds of cultivation? In no sane man's case should other men's ideas be added in such quantity and variety as to choke the original seed. And will every individual man learn this also: that without a purpose steadily carried out, or with only a flickering and occasional purpose, the final results of his life and work can bear no more proportion to his real powers and duties than the work of the habitual or occasional drunkard can to his? Nowhere in the world is constant equilibrium so important to a man as in the affairs of his own mind.

